# High-efficiency and low-energy ship recognition strategy based on spiking neural network in SAR images

**DOI:** 10.3389/fnbot.2022.970832

**Published:** 2022-09-02

**Authors:** Hongtu Xie, Xinqiao Jiang, Xiao Hu, Zhitao Wu, Guoqian Wang, Kai Xie

**Affiliations:** ^1^School of Electronics and Communication Engineering, Shenzhen Campus of Sun Yat-sen University, Shenzhen, China; ^2^The Fifth Affiliated Hospital, Guangzhou Medical University, Guangzhou, China

**Keywords:** ship target recognition, synthetic aperture radar (SAR), SAR image, spiking neural network (SNN), high-efficiency, low-energy

## Abstract

Ship recognition using synthetic aperture radar (SAR) images has important applications in the military and civilian fields. Aiming at the problems of the many model parameters and high-energy losses in the traditional deep learning methods for the target recognition in the SAR images, this study has proposed a high-efficiency and low-energy ship recognition strategy based on the spiking neural network (SNN) in the SAR images. First, the visual attention mechanism is used to extract the visual saliency map from the SAR image, and then the Poisson encoder is used to encode it into a spike train, which can suppress the background noise while retaining the visual saliency feature of the SAR image. Besides, an SNN model integrating the time-series information is constructed by combining the leaked and integrated firing spiking neurons with the convolutional neural network (CNN), which can use the firing frequency of the spiking neurons to realize the ship recognition in SAR images. Finally, to solve the problem that SNN model is difficult to train, the arctangent function is used as the surrogate gradient function of the spike emission function during the backpropagation. Hence, applying this backpropagation method to the training process can optimize the SNN model. The experimental results show the following: (1) the proposed strategy can more accurately recognize the ship in the SAR image, and the F1 score can reach 98.55%, which has a better recognition performance than the other traditional deep learning methods; (2) the proposed strategy has the least amount of model parameters (only 3.11MB), which is far less than the model parameters of the other traditional deep learning methods; (3) the proposed strategy has fewer operations (only 17.97M) and can reach 1/30 time of operands of the other traditional deep learning methods, which shows the high efficiency of the proposed strategy using the spike emission signals; (4) the proposed strategy has the energy loss of 1.38 × 10^−6^J, which can achieve the low energy advantage of nearly three orders of the magnitude compared to the other traditional deep learning methods, indicating that the proposed strategy has a significant energy efficiency.

## Introduction

Synthetic aperture radar (SAR) is a high-resolution microwave imaging and detection system (Xie et al., 2016, 2017, 2020; Zhang et al., 2022), which has the advantage of all-weather, all-day, and harsh environment work; thus, it can observe the land and ocean in real time for a long time (López-Randulfe et al., 2021; Yu et al., 2022). In recent years, related technology research on ship identification using SAR images has received great attention in the field of marine remote sensing (Lang et al., 2022; Wang et al., 2022). However, the traditional SAR target recognition methods mainly rely on artificially designed features, which are susceptible to complex background interferences and have shortcomings, such as poor recognition accuracy, low recognition efficiency, and weak generalization ability. The deep learning method represented by the neural network can learn the image features independently without relying on the manual design. It has the characteristics of a high degree of automation and strong recognition capability, which has made breakthroughs in the task of target recognition in SAR images (Song et al., 2022; Yang and Lang, 2022).

With the continuous development of the deep learning technology, the computing power of the model has been greatly improved, but it also faces a key question regarding the computational cost and energy consumption involved in the neural network. According to a recent study published by the University of Massachusetts (Strubell et al., 2019), the training process of the deep learning models is expensive, and this problem will become more and more serious when the computing power of the model increases. The increase in the energy consumption in artificial intelligence (AI) computing costs is first attributed to the emergence of the increasingly complex AI models. In 2018, the natural language processing model, BERT released by Google has reached 300 million parameters (Devlin et al., 2019). In 2020, the latest GPT-3 model released by the OpenAI researchers has 175 billion parameters (Brown et al., 2020). In 2021, Google has launched the larger language model, Switch Transformer with 1.6 trillion parameters (Fedus et al., 2021). Although the design of the artificial neural network (ANN) is inspired by the human brain and has been widely borrowed from neuroscience, it is fundamentally different from the biological neural networks and cannot completely imitate the operation mechanism of the neurons in the brain; therefore, the efficiency is far from being comparable to that of the human brain.

Although the traditional ANN has made breakthroughs in many tasks, such as recognition, detection, and segmentation (Zhu et al., 2021), the energy-consumption problem has limited its wider deployment and application. To solve this problem, the third generation ANN called the spiking neural network (SNN) was proposed. The SNN based on the brain-like computing framework uses the spiking neurons as the basic computing unit and transmits the information through sparse spike trains, which is called a new generation of green AI technology with lower energy consumption. The SNN was first proposed by Maass (1997), inspired by the operating mechanism of the biological neurons. The main core idea is to use the spike trains and spike functions to simulate the process of information encoding and information transmission between the biological neurons. The SNN can more accurately imitate the information expression and the processing ability of the human brain, which is a brain-like computing model with high biological plasticity, event-driven characteristics, and low energy consumption (Roy et al., 2019). Based on the cognitive level of the human brain and the related theories of neuroscience, the SNN constructs the spiking neurons through the biological visual system and biological neuron computing mechanism. It has the characteristics of being closer to biological reality, so it can better simulate the complex system of the biological brain, and it is a new generation of more efficient and intelligent AI systems.

At present, research on the SNN mainly focuses on the field of computer vision based on optical images. Fang et al. (2021a) have proposed a spiking neuron-based residual network to solve the image classification problems by adding the SNN neuron layers between the traditional residual units. Cui et al. (2012) have proposed linear coding and non-linear coding methods based on the time-to-first-spike coding strategy, which converts the gray value of the image pixels into discrete spike trains for image segmentation. Kim et al. (2019) applied the deep SNN to the field of target detection for the first time, and then proposed a detection model based on the SNN (Spiking-YOLO). This method provides a faster and more accurate message passing between the neurons by using the techniques, such as channel-wise normalization and signed neurons with unbalanced thresholds, which can achieve better convergence and lower energy consumption than the ANN models.

To solve the problems of many model parameters and high energy consumption in the traditional neural network for the SAR target recognition, this study extends the SNN to the ship recognition in SAR images. First, we have to find how to encode the SAR image into the time-correlated spike train, i.e., how to convert the pixels in SAR images into a spike train that the SNN can understand. Then, it is necessary to build an SNN model that can effectively extract the features from SAR images. Finally, considering the difficulty of the training caused by the discrete characteristics of the SNN and the spatiotemporal correlation, an effective learning algorithm is needed to be designed for the training. In view of the above problems, this study focuses on the SNN coding, model construction, and training, and proposes a high-efficiency and low-energy ship recognition strategy based on the SNN in SAR images, which is called the SpikingSAR. Section Spike encoder based on visual attention mechanism presents the spike encoder based on the visual attention mechanism, and an SNN model integrating the time-series information is constructed in Section SpikingSAR model. In Section SpikingSAR model, the arctangent function is used as the surrogate gradient function, and the corresponding loss function is designed. The experimental results are shown in Section Experiment and discussion. A conclusion is given in Section Conclusion.

## Ship recognition strategy

**Figure 1:**
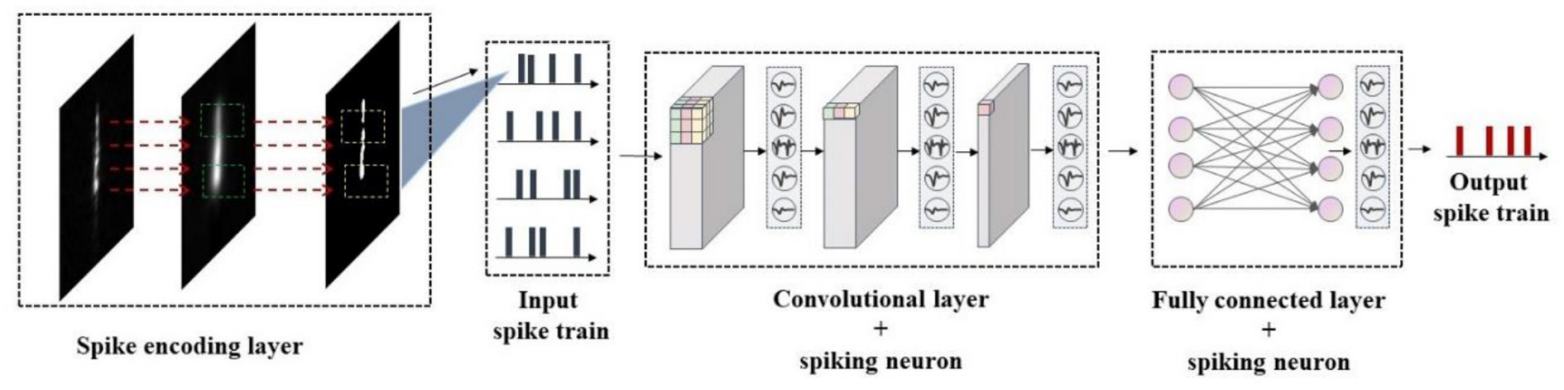
Model framework of the proposed SpikingSAR algorithm.

In this study, a high-efficiency and low-energy ship recognition strategy based on the SNN in the SAR images has been proposed, whose model framework is shown in Figure 1. First, the visual attention mechanism is used to extract the visual saliency map from the SAR image, and then the Poisson encoder is used to encode it into a spike train, which can suppress the background noise while retaining the visual saliency feature of the SAR image. Besides, an SNN model integrating the time-series information has been constructed by combining the leaked and integrated firing spiking neurons with the convolutional neural network (CNN), which can use the firing frequency of the spiking neurons to realize the ship recognition in the SAR image. Finally, to solve the problem that the SNN model is difficult to train, the arctangent function has been used as the gradient replacement function of the spike emission function during the backpropagation to calculate the gradient, and thus applying this backpropagation algorithm to the training process can further optimize the SNN model. The ship recognition results in the real SAR images show that the proposed strategy can achieve better performance than the traditional recognition methods and achieve comparable or even better performance than the CNN methods, which can accurately identify the ship in the complex SAR images. In addition, the proposed strategy uses discrete spike trains to transmit the information, which has fewer parameters and lower energy consumption compared with the traditional deep learning methods, so it is helpful for deployment on the small terminal components.

### Spike encoder based on visual attention mechanism

The SNN uses the spike as the basic information transmission unit, and the input is the spike train to represent the encoding of the specific input data. Given that the image data are mostly continuous and static real-valued values, it is difficult to represent the discrete time-correlated spike trains; thus, the additional encoding of the input is required. A commonly used method of encoding still image data is the rate-based encoding, which converts the input image into a mapping of spikes at each time step. Given a simulation duration *T*, a sequence of Poisson spikes is sampled from each pixel according to a suitable distribution, with a spike emission probability proportional to the pixel grayscale intensity. This coding method can not only preserve the integrity of the input image but also can binarize the data in the time domain. This has received high attention and wide application in many fields (Fang et al., 2021a; Fedus et al., 2021).

Provided that the gray value of the input image is between 0 and 1, the pixel value distribution of the SAR image is quite different from that of the optical image, and it is difficult to obtain an effective spike train by directly performing the rate-based spike coding method. In addition, due to the special natural environment at the sea (such as the sea fog, etc.) and various types of noise in the SAR images, certain preprocessing is required to filter and identify a large amount of interference information, and the ship in the SAR image can be accurately obtained only after removing the noise information. The traditional method first filters the image to reduce the influence of the noise on the SAR image. However, the filtering will lead to the widening of the image edge and the loss of the positioning information of the edge, which may easily miss to detect smaller ships (Zhang and Hu, 2017). Considering that the concerned ship only occupies a small part of the SAR image, and most of the areas are marine backgrounds, if the more concerned areas in the SAR image can be pre-selected and then re-encoded according to the importance of each area, it will help to improve the accuracy rate of the model of the detection model, which accelerates the convergence rate of the model during the training process. Therefore, this study proposes a spike encoder based on the visual attention mechanism (Li et al., 2016), which can preferentially focus on the salient regions with the obvious visual features in the SAR image, and ignore the background clutter noise, thereby reducing the identification range of the small ships.

#### Visual attention model

**Figure 2:**
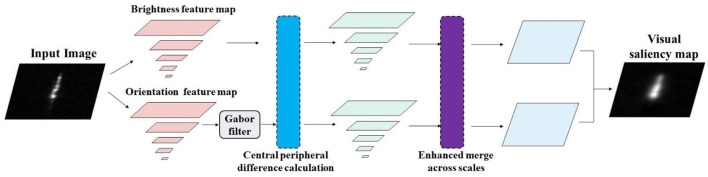
Visual attention model architecture.

The visual attention mechanism is an unsupervised image processing method that does not need to rely on the prior knowledge or cognitive assumptions of the images, which can prioritize the target area of interest. Compared with the traditional methods based on image statistical modeling, the visual attention method has a faster running speed and stronger robustness. In this study, the visual attention mechanism model is applied to the SAR images, focusing on the brightness information and orientation information in the SAR images. The structure of the visual attention model is shown in Figure 2.

(1) Brightness information extraction. Given an input image *J*, the Gaussian pyramid is first used to extract the brightness feature map with a resolution of two, and an eight-level down-sampling process is realized in which the scale of the SAR image is gradually decreased in the horizontal and vertical directions. The brightness feature map of each image layer is *I*(*k*), where *k* ∈ [0,8] represents different levels in the Gaussian pyramid structure of the images. There is a strong correlation between the adjacent pixels in the image at the texture and gray level. If a pixel is more different from the surrounding pixels, it is easier to attract the visual attention and becomes a visually salient point. Thus, the center-peripheral difference method is introduced to further process the feature maps of the different scale resolutions to obtain the attention information. Define ⊖ as the central peripheral difference operator; therefore, the extraction of the brightness feature maps is to scale the feature maps of the different levels to the same scale and then perform the pixel-by-pixel subtraction, which is given by:


(1) $$\begin{array}{l} I\left(c,s\right)=|I\left(c\right)⊖I\left(s\right)| \end{array}$$

where, c ∈ {2,3,4}, s = c+δ, and δ ∈ {3,4}. The different levels of the attention information are obtained by performing the central-peripheral difference operation on the feature map.

(2) Orientation information extraction. The process of orientation information extraction is similar to that of brightness information extraction. First, the Gaussian pyramid is used to down-sample the input image at the eight levels to obtain a multi-scale feature map, and then further orientation feature extraction is performed. Gabor filter is an effective method to extract the local features of the image space. In this paper, the two-dimensional Gabor filter is used to extract the orientation channel feature information of the images, which is given by:


(2) $$\begin{array}{l} G\left(x,y\right)=\frac{1}{2\pi \sigma_{x}\sigma_{y}\;}\exp\left[-\pi \left(\frac{{\left(x-x_{0}\right)}^{2}}{\sigma_{x}^{2}}+\frac{{\left(y-y_{0}\right)}^{2}}{\sigma_{y}^{2}}\right)\right]\\ \exp\left[i\left(\xi_{0}x+\upsilon_{0}y\right)\right] \end{array}$$

where, (*x*_0_, *y*_0_) is the coordinate position of the target center in the image, (ξ_0_, υ_0_) is the optimal spatial frequency of the filter in the frequency domain, and *i* is the imaginary unit. $\sigma_{\text{x}}^{2}$ is the variance of the Gaussian function in the *x* axis direction, $\sigma_{\text{y}}^{2}$ is the variance of the Gaussian function in the *y* axis direction, and $\sigma_{\text{x}}^{2}$ and $\sigma_{\text{y}}^{2}$ determine the size of the acceptable region of the Gabor filter kernel. The orientation feature extraction is performed on the feature map of each level in the Gaussian pyramid, which can obtain the orientation feature maps of the different scales *O*(*k*), where *k* ∈ [0,8] represents the different levels in the image Gaussian pyramid structure. Then, the attention information of the orientation feature is extracted by using the center-periphery difference method, which is:


(3) $$\begin{array}{l} O\left(c,s,\theta \right)=|O\left(c,\theta \right)⊖O\left(s,\theta \right)| \end{array}$$

where θ is the four directions of the Gabor filter, θ ∈ {0°, 45°, 90°, 135°}.

(3) Globally enhanced merging. Through the above feature extraction, a series of brightness feature maps and orientation feature maps of the different scales are obtained. Since the detection and recognition of the targets in SAR images usually lack the target prior information, and the visual attention method is an unsupervised method, this study adopts the global enhanced merging strategy to fuse the attention information of the brightness and orientation feature maps to obtain the visual saliency map. The globally enhanced merging is a feature information merging strategy that does not require the target prior information, which can effectively enhance the saliency peak region in the feature map, thereby removing the background clutter. The method mainly consists of three steps. First, normalize the feature map to [0, *N*], N ∈ [0, 255] limits the normalization range. Second, calculate the global maximum *M* and local average m^−^. Finally, multiply the weight (M–m^−^)^2^ for each feature graph. If *N* represents the global enhanced merging process, the merging process of the brightness channel saliency map fused with the central peripheral difference operator is given by:


(4) $$\begin{array}{l} I=\overset{4}{\underset{c=2}{\sum }}\overset{c+4}{\underset{s=c+3}{\sum }}N\left(I\left(c,s\right)\right) \end{array}$$

Similarly, the merging process of the orientation channel saliency maps is expressed as:


(5) $$\begin{array}{l} O=\underset{\theta \in 0^{{}^{\circ}},45^{{}^{\circ}},90^{{}^{\circ}},135^{{}^{\circ}}}{\sum }N\left(\overset{4}{\underset{c=2}{\sum }}\overset{c+4}{\underset{s=c+3}{\sum }}N\left(O\left(c,s,\theta \right)\right)\right) \end{array}$$

Finally, according to the work proposed by Li et al. (2016), the global merging strategy is applied to the Equations (4) and (5) to obtain a visual saliency map *S*; thus, the merging process is expressed as:


(6) $$\begin{array}{l} S=\frac{1}{2}\left(N\left(I\right)+N\left(O\right)\right) \end{array}$$

Due to the normalization operation adopted by this strategy, the final visual saliency map *S* ∈ [0,1] meets the preconditions for the spike coding in this study. In addition, due to the larger gray value of the region with more prominent visual features, the corresponding encoded spike train in this region has a greater probability of transmitting the spike information.

#### Poisson encoder

**Figure 3:**
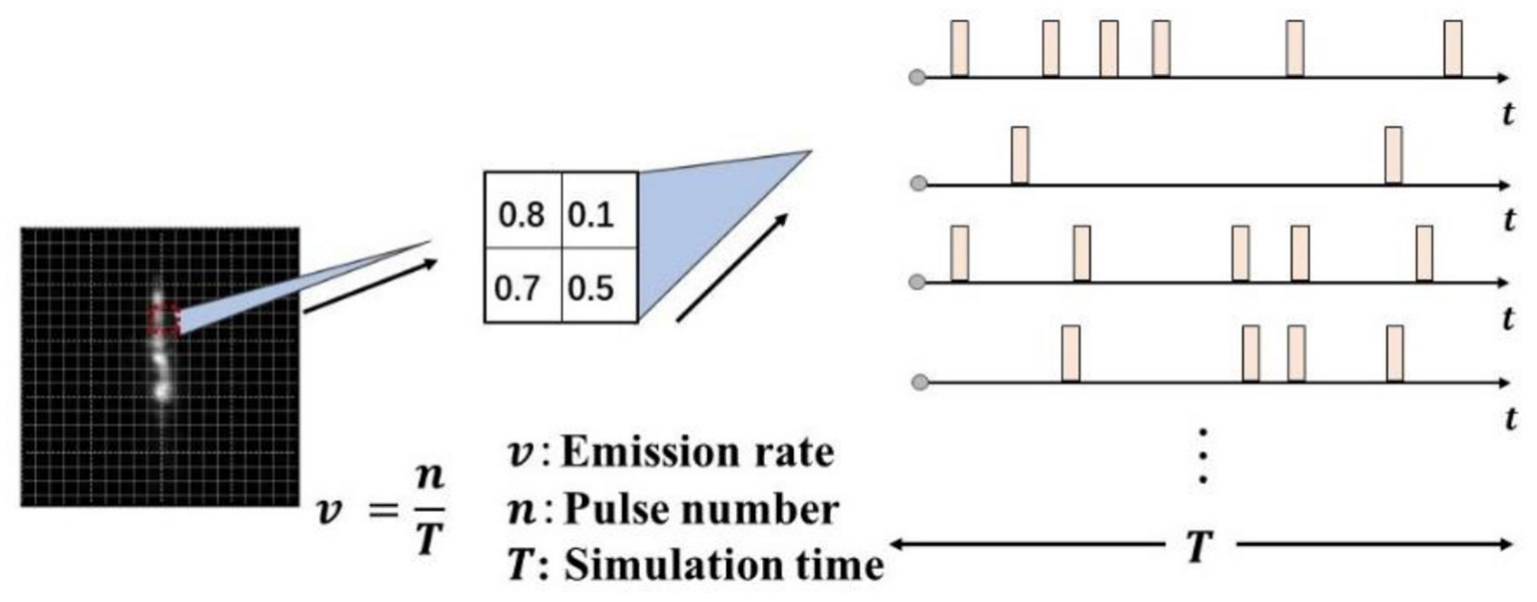
Schematic diagram of Poisson encoding process.

In the SNN, the encoder mainly converts the continuous real-valued signal of the input into a discrete spike train with time information and preserves most of the information of the data as much as possible. In this study, a rate-based Poisson encoder is used, which can encode the input real-value data into the spikes whose firing number distribution conforms to the Poisson process and is widely used in the spike train estimation and neural network background noise simulation. In the rate-based Poisson coding, for the input *x* ∈ [0,1], within one simulation step, the probability of the spike emission is set as *p* = *x*, then the sampling process of the Poisson coding can be given by:


(7) $$\begin{array}{l} \hat{x}=\{\begin{array}{ll} 1 & \text{if }x\;⩾\;p\\ \; & \text{ subject to p}\in \text{U}(\text{0},\text{1})\\ 0 & \text{otherwise} \end{array} \end{array}$$

where U (0, 1) is a uniform distribution. Considering the time correlation of the spike trains, this study additionally introduces the simulation duration variable *T* and applies the Poisson coding process at each time step to convert the input of the static pixel data into the mapping of the spike train on each time step. As shown in Figure 3, each pixel generates *T* spike trains within *T* time steps, and the emission probability of the spike is proportional to the size of the pixel value.

### SpikingSAR model

The time-driven SNN is a network with Markov properties, and the output at the current moment is only related to the output at the previous moment and the current state of neuron membrane voltage. The input of the SNN is the voltage increment at the current moment, which represents the charging process of the neuron potential. The output value is a discrete value of 0 or 1, which represents whether the spike is fired or not and is the discharge process of the neuron potential. The neurons have a refractory period for a short time after firing; that is, they do not respond to the external input signals. To simulate this process, a reset process was additionally introduced after the neuron is fired and the voltage was reset to keep the neuron in a resting state. Therefore, a typical SNN neuron can be described by three processes of charging, discharging, and resetting.

#### Leaky integrate-and-fire neuron model

The leaky integrate-and-fire (LIF) model (Gerstner et al., 2014) is an improvement on the integrated firing spiking neuron model. The LIF model takes into account another physiological factor: the cell membrane is not a perfect capacitor, and the charge slowly leaks through the cell membrane over time, allowing the membrane voltage to return to its resting potential. The LIF model regards the electrical properties of the cell membrane as a parallel combination of resistance and capacitance. Compared with the integrated firing model, the LIF model has better biological reliability and biological accuracy, which can more accurately describe the state changes of the neurons.

For the LIF neurons, it can be described by a differential equation:


(8) $$\begin{array}{l} C\frac{dV\left(t\right)}{dt}=-\frac{1}{R}\left(V\left(t-1\right)-V_{reset\;}\right)+I\left(t\right) \end{array}$$

where, *V*(*t*) is the membrane voltage of the neuron at the time *t*, *R* is the membrane resistance, *C* is the capacitance, *V*_reset_ is the resting voltage or equilibrium voltage of the neuron, and *I*(*t*) represents the input current at the *t* time. The neurons are often biologically simulated using the discrete forms. To ensure the availability of the SNN model calculation, a discrete form is used to approximate the differential expression, which is given by:


(9) $$\begin{array}{l} \tau \left(V\left(t\right)-V\left(t-1\right)\right)=-\left(V\left(t-1\right)-V_{reset\;}\right)+X\left(t\right) \end{array}$$

where, τ = *RC* is the membrane time constant and *X*(*t*) = *I*(*t*)*R* is the external input at the *t* time. Therefore, the charging equation corresponding to the LIF model is:


(10) $$\begin{array}{l} f\left(V\left(t-1\right),X\left(t\right)\right)=V\left(t-1\right)+\frac{1}{\tau }\left(-\left(V\left(t-1\right)-V_{\text{reset}}\right)+X\left(t\right)\right) \end{array}$$

where, *f* is the state update function of the neuron charging moment. The discharge process of the LIF model is similar to that of the integrated firing model. When the charge accumulates to a certain level, that is, when the membrane voltage reaches the threshold *V*_t*h*_, the neuron fires the spikes. The spike emission can be described by the Heaviside step function, which is given by:


(11) $$\begin{array}{l} \Theta (x)=\{\begin{array}{cc} 1 & if\;x\;⩾\;0\\ 0 & \text{otherwise} \end{array} \end{array}$$

The reset process of the LIF model occurs after the firing of the spike, which consumes the previously accumulated charge of the neuron, so there is a momentary decrease in the membrane potential. In general, there are two types of the hard reset and soft reset:


(12) $$\begin{array}{l} V(t)=\{\begin{array}{cc} V_{\text{reset}} & \text{Hard}\\ V(t)-V_{\text{reset}} & \text{Soft} \end{array} \end{array}$$

Considering that when the membrane potential does not reach the threshold, the neuron does not emit the spikes and the potential remains unchanged, so the spike reset process of the LIF model is given by:


(13) $$\begin{array}{l} g\left(H\left(t\right),S\left(t\right),V_{\text{reset}}\right)=H\left(t\right)\left(1-S\left(t\right)+V_{\text{reset}}S\left(t\right)\right) \end{array}$$

where, *g* is the reset function after the spike is fired, *H*(*t*) is the hidden state of the neuron at the *t* time, and *S*(*t*) is the spike firing state of the neuron.

#### Backbone network

The charging process of the spiking neurons requires receiving an exogenous input *X*(*t*), accumulating the charges such that the membrane voltage reaches a threshold for firing the spikes. The widely used ANN is very suitable for simulating a continuous signal input, so this study combines it with the spiking neuron model as the backbone network for the image recognition. The process of simulating the input voltage of the neuron *j* using the ANN can be expressed by:


(14) $$\begin{array}{l} X^{l}{\left(t\right)}_{j}=\sum_{i}w_{ij}^{l}s_{i}^{l-1}+b_{j}^{l} \end{array}$$

where, $s_{i}^{l-1}$ is the input spike train of the (*l* − 1)-th layer, $w_{ij}^{l}$ and $b_{j}^{l}$ are the weights and biases of the *l*-th layer, respectively, and $X^{l}{\left(t\right)}_{j}$ is the input voltage of the *l*-th layer. The widely used ANNs are mainly the CNN and fully connected networks (FCN), both of which can be used as the models for simulating the input voltage of the SNN. Given that the input spike train is encoded by the visual saliency map of the SAR image, the use of the CNN is more able to capture the local spatial information of the input and has the advantages of the less parameters, and then a full connection is introduced at the end of the CNN layer, which can integrate the feature information extracted by the convolution layer to classify the image. Thus, the CNN and FCN are alternately stacked with the LIF spiking neurons as the backbone network of the proposed SNN model.

In addition, the ANN is often combined with the batch normalization layers, dropout layers, pooling layers, and activation function layers. The batch normalization layer can normalize the input data of each layer without losing the important information as much as possible, so that the data distribution is relatively stable, and the learning speed of the model is accelerated. The batch-normalized data conforms to a standard Gaussian distribution and is usually located in the non-gradient saturation region of the activation function, thus avoiding the vanishing gradient problem. The dropout layer can randomly drop the neurons with a certain probability, thereby reducing the interaction between the nodes in the hidden layer of the model and avoiding the model overfitting. The role of the pooling layer is to down-sample the feature map and to gradually reduce the size of the feature space, which can avoid the high computational complexity of the network and enhance the spatial invariance of the network. Commonly used pooling methods are the average pooling and max pooling. The existing research shows that using the max pooling in the SNN loses the effective information (Cheng et al., 2020), so average pooling is widely used. However, Fang et al. (2021b) believed that the max pooling is consistent with the temporal information processing capability of the SNN, which can improve the SNN ability to fit the time series data while reducing the computational cost of the next-layer network.

**Figure 4:**
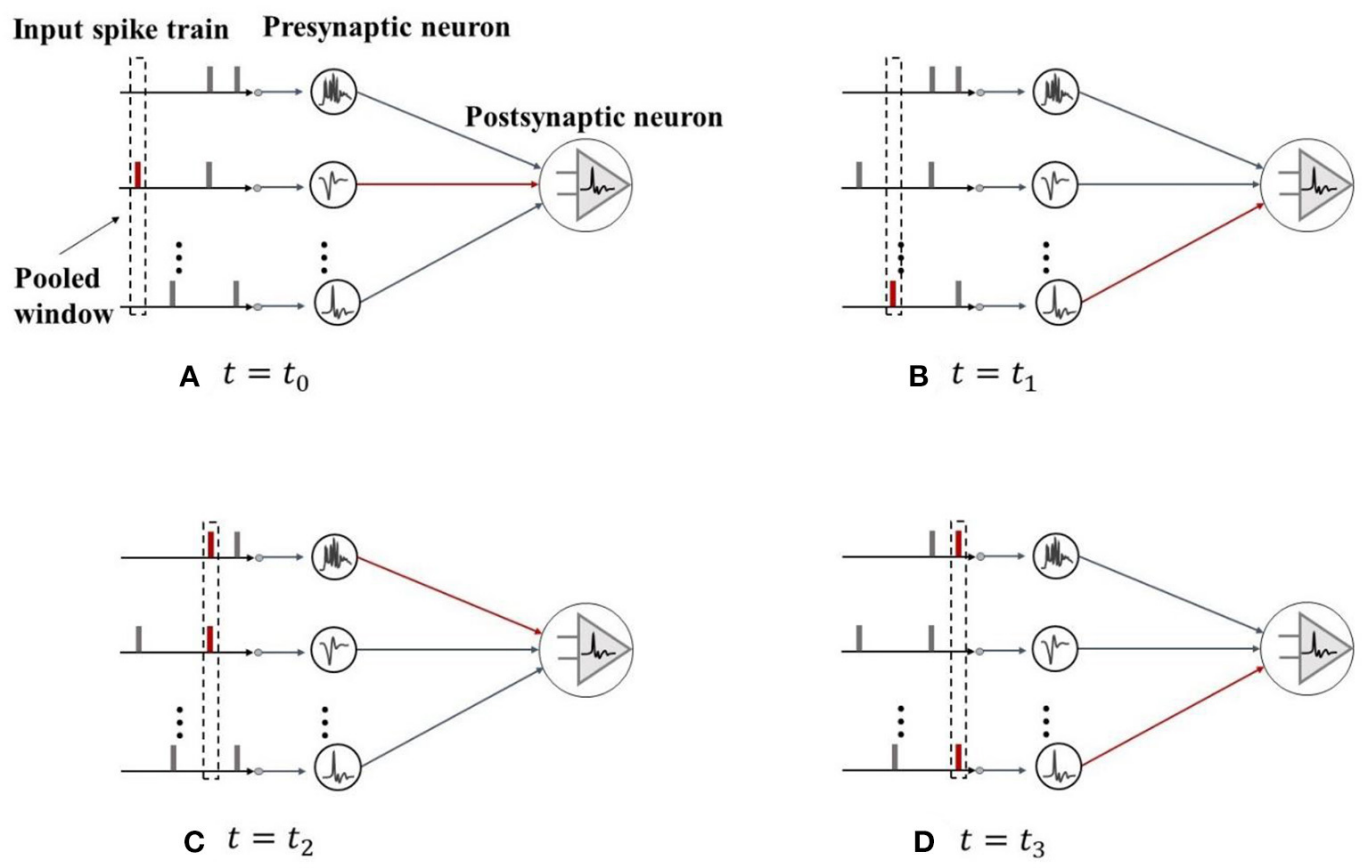
Schematic diagram of the spike max pooling. **(A,B)** In each time step, only the most active neurons in the pooling window can communicate with the neurons in the next layer. **(C,D)** When two or more neurons fire the spikes at the same time, one of the neurons is randomly selected for the messaging.

Therefore, this study adopts the spike max pooling method as an improved pooling layer, as shown in Figure 4. Unlike the average pooling which transmits the information to the next layer of the neurons evenly, the spike max pooling introduces a “winner takes all” mechanism, where in each time step, only the most active neurons in the pooling window can communicate with the neurons in the next layer, and other neurons in the pooling window are ignored. The spike max pooling layer can dynamically adjust the connections between the neurons to improve the neuron capability to emit the spikes, thereby enhancing the SNN ability to fit the time series data. In addition, the output of the spike max pooling layer is still the binary data, which can maintain the discreteness of the spike train compared to the average pooling of output floating point numbers and use logical operations instead of matrix multiplication operations, thereby improving the operation speed and reduce the energy consumption. The most important feature of the ANN is that it can approximate the non-linear functions arbitrarily, and this feature largely benefits from the existence of the non-linear activation functions. Different from the traditional ANN, due to the existence of the Heaviside step spike emission function, the SNN that introduces the spiking neuron itself has a non-linear nature. Thus, the activation layer can be directly replaced by the spiking neuron, and the activation function is replaced by the spike emission function, which further simplifies the computational complexity of the SNN model.

**Figure 5:**
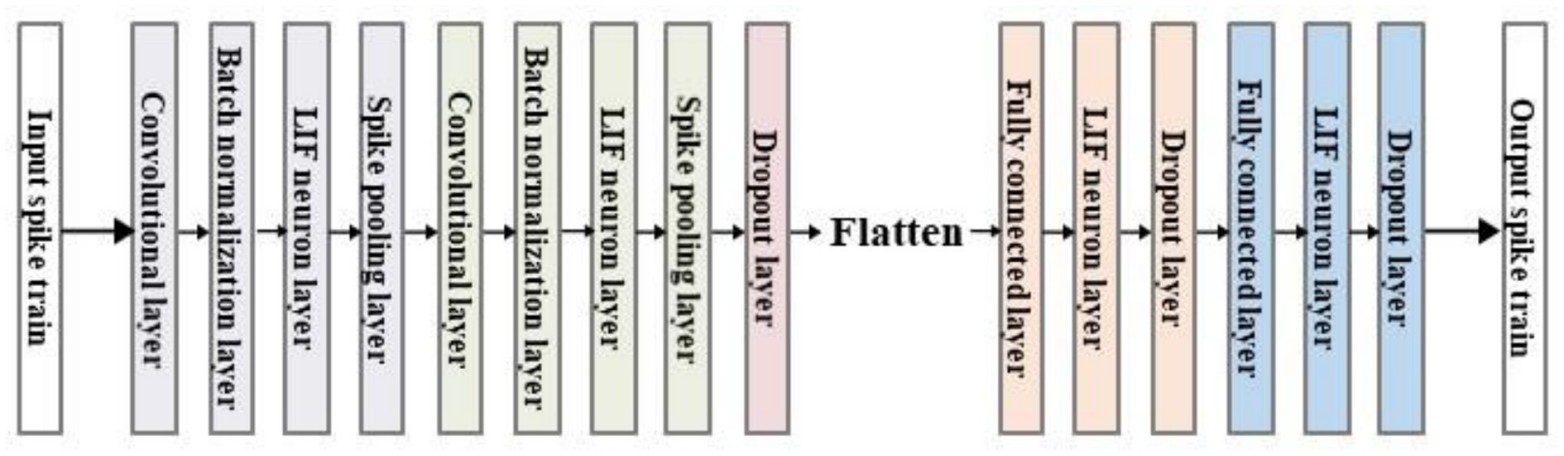
Structure diagram of the backbone network.

The backbone network used in this paper is shown in Figure 5. The network structure draws on the model of the traditional CNN, combining the convolutional layer, batch normalization layer, pooling layer, dropout layer, and the final FCN. The input of the network is the encoded spike signal, and the output is the firing spike of each neuron in *T* time steps.

#### Model training

The activation functions used by the traditional ANN are the differentiable activation functions, such as the ReLU, Sigmoid, and Tanh functions. However, the spiking emission function Θ(*x*) of the SNN is usually non-differentiable, which makes it impossible to use gradient descent and backpropagation algorithms for the training optimization even though the SNN is structurally very similar to the ANN.

This study adopts the method of training with the surrogate gradients by approximating the spike function with a gate function σ(*x*) which is very similar in the form to Θ(*x*) but differentiable, and then computes the approximate surrogate gradient for the SNN to update. The surrogate gradient methods have been shown to be very effective in training SNN, and the models trained with this method can achieve the comparable performance to ANNs on many tasks Fang et al. (2021b). The core idea of the surrogate gradient method is: (1) in the forward propagation, the spike emission function Θ(*x*) is still used as the gating function to activate the spiking neuron and fire the spike when the voltage reaches the threshold; (2) in the backpropagation, the surrogate function is used to calculate the approximate gradient, and the surrogate gradient is used to optimize the update of the network.

A common surrogate function is the smoothed Sigmoid function σ(α*x*) = (1 + *exp*(–α*x*))^−1^, where α is a smooth factor. The factor can control the smoothness of the function. The larger the value of α, the smoother is the function, and thus the more approximate is the spike emission function Θ(*x*). However, it is easier to explode the gradient when it is close to the origin and disappears when it is far away from the origin, making the network more difficult to train. Correspondingly, the gradient of the Sigmoid function calculated during the backpropagation is σ(α*x*)′ = α · σ(α*x*) · (1 – σ(α*x*)).

However, the Sigmoid function cannot fit the Heaviside step spike emission function well, and when α is large, the Sigmoid function is prone to saturation at both ends, causing the problem of the gradient disappearance and increasing the difficulty of the network training. To solve the above problems, this study proposes to use the smoothed arc tangent function (Arctan) as the gradient replacement function during the model training. In the forward propagation, the computation of the smoothed Arctan is:


(15) $$\begin{array}{l} \sigma \left(x\right)=\frac{1}{\pi }\arctan\left(\frac{\pi }{2}\alpha x\right)+\frac{1}{2} \end{array}$$

During the backpropagation, the gradient of the smoothed Arctan function is computed as:


(16) $$\begin{array}{l} \sigma^{\prime }\left(x\right)=\frac{\alpha }{2\left(1\text{+}{\left(\frac{\pi }{2}\alpha x\right)}^{2}\right)} \end{array}$$

**Figure 6:**
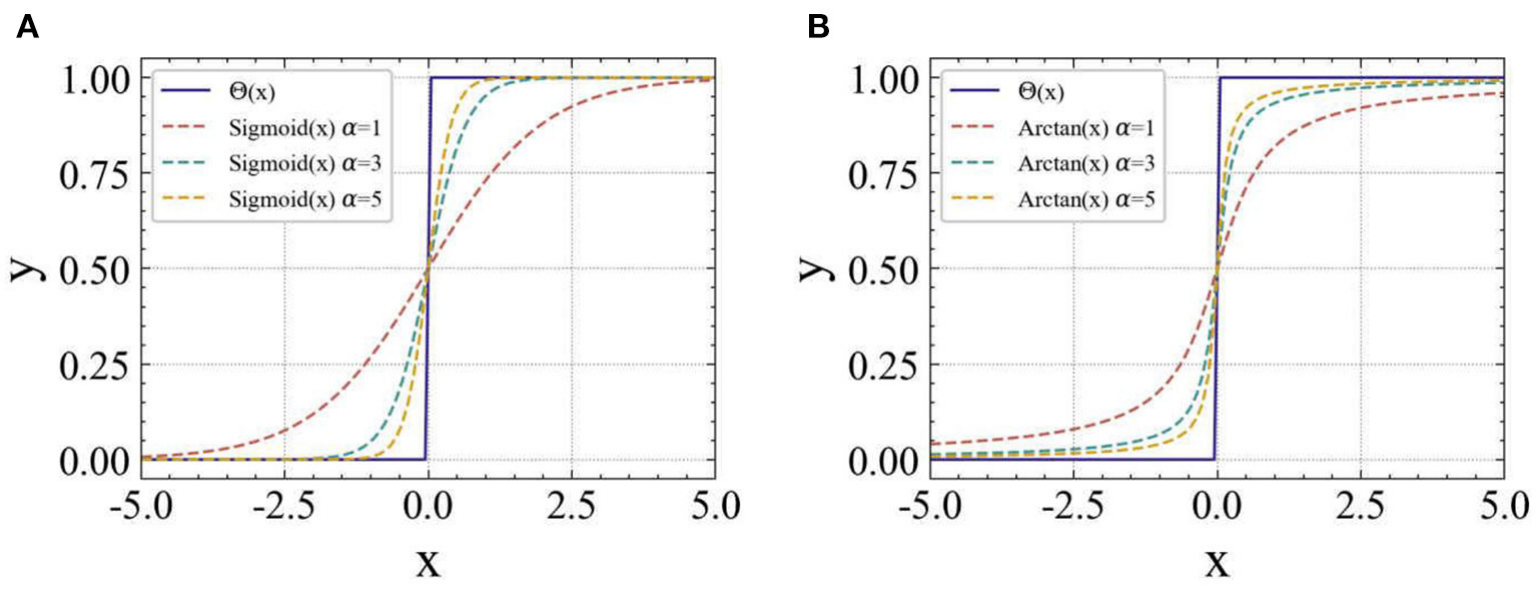
Comparison of the surrogate gradient function and Heaviside step function Θ(*x*) for the different values of α. **(A)** Smoothed Sigmoid function, **(B)** Smoothed Arctan function.

Figures 6A,B, respectively, show the comparison of two different surrogate gradient functions with the Heaviside step spike emission function Θ(*x*) at different α values. It can be found that the Arctan function is more similar to the Heaviside step function in form, and as the value of α increases, the gradient disappears less easily than the Sigmoid function.

To train an SNN with the backpropagation method, an appropriate loss function needs to be designed. Provided that the number of the categories of the SAR image is *C*, the output spike train is *O*=[*o*_t,*i*_] ∈ R^C × *T*^, and the corresponding category matrix is *Y* = [*y*_t,*i*_] ∈ R^C × *T*^. If the true label of an image is *l*, then the neuron whose final output represents the class *l* should have the highest level of the excitation (i.e., fire the most spikes), and the rest of the neurons should remain inhibited (i.e., fire fewer spikes). Therefore, this study adopts the mean square error function as the loss function for the model training. The definition of this loss function can be defined by:


(17) $$\begin{array}{l} L\left(O,Y\right)=\frac{\text{1}}{T}\overset{T-1}{\underset{t=0}{\sum }}\frac{1}{C}\overset{C-1}{\underset{i=0}{\sum }}{\left(O_{t,i}-y_{t,i}\right)}^{2} \end{array}$$

where, *y*_*t,i*_ is 1 if only if *i* = *l*, and the rest are zero. When the model training converges, the recognition and prediction category *l*_p_ of the input image is the category represented by the neuron with the largest number of firing the spikes, which is given by:


(18) $$\begin{array}{l} l_{p}=\arg\underset{i}{\max}\frac{\text{1}}{T}\overset{T-1}{\underset{t=0}{\sum }}O_{t,i} \end{array}$$

## Experiment and discussion

### Experimental setting

**Figure 7:**
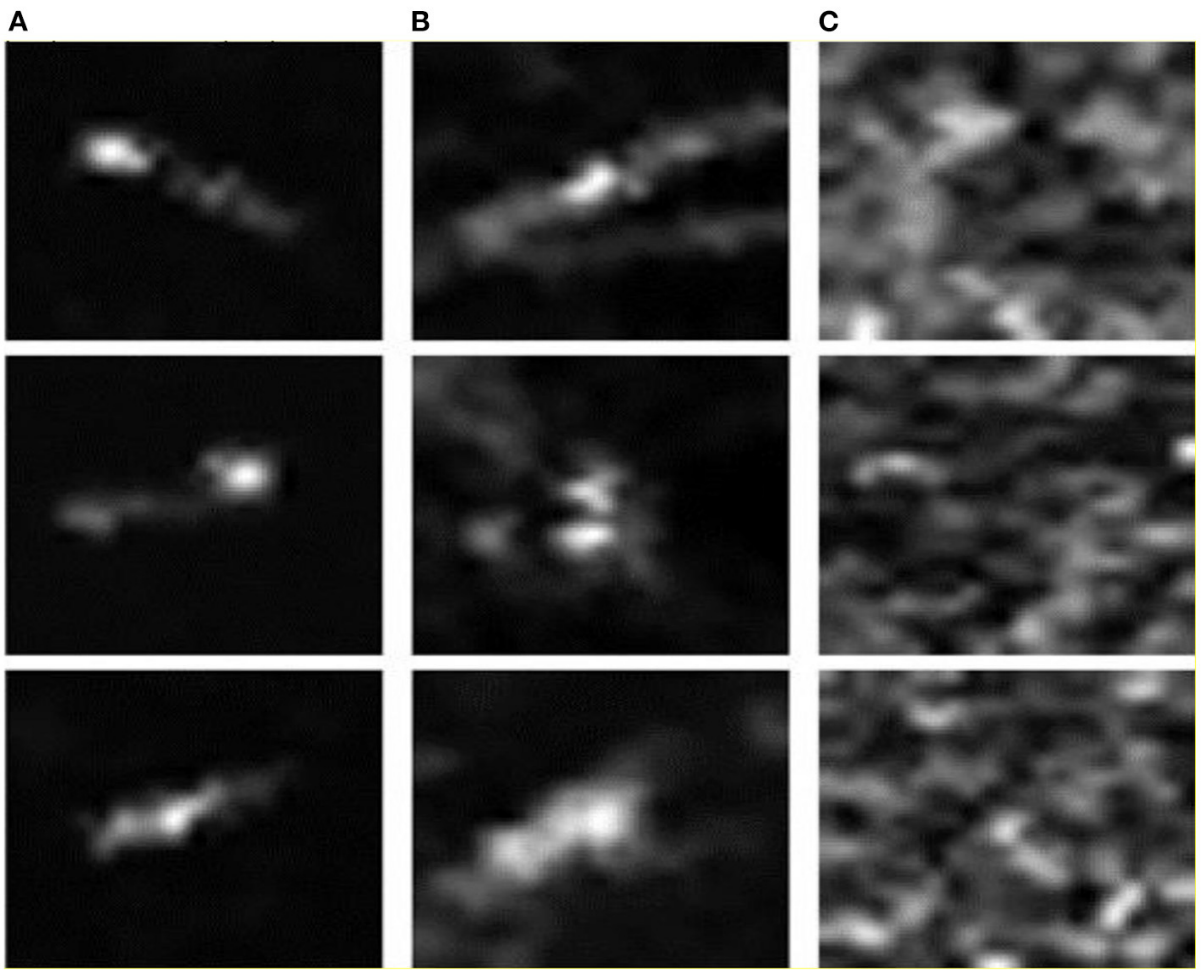
Nine examples of the three types of sub-images in the SAR-Dataset. **(A)** Ship images; **(B)** Ship-like images; **(C)** Ocean background images.

To better verify the correctness and effectiveness of the proposed method, this study uses the real SAR ship datasets for the experiment, i.e., the SAR-Dataset. The SAR-Dataset is a dataset for ship recognition and detection published by Schwegmann et al. (2016). It is constructed from 22 images collected by the Sentinel-1 satellite and 3 images collected by the RADARSAT-2 satellite, containing a total of 42 dual-polarization and 4 single–polarization radiometric calibration images. The dataset covers ~80% of the exclusive economic zone in South Africa, which includes the multiple high-vessel density port scenarios. After the analysis and processing, the dataset contains three types of image samples, includes the ships, ship-like images (false alarm images), and ocean background images. Among them, the ship category is a positive sample, while the rest of the categories are used as the negative sample images. The dataset has the same number of the samples for each category, which contains 1,596 SAR images of the size 75 × 75. Moreover, the sample distribution of the SAR-Dataset is balanced, which is conducive to the model training and evaluation. Figure 7 shows nine examples of the three types of the sub-images (ship images, ship-like images, and ocean background images) in the SAR-Dataset. It is seen that the ship-like images are very similar to the ship images, which undoubtedly poses a huge challenge to the recognition model.

To fully verify the effectiveness of the proposed method, this study uses two types of methods for comparison, including the traditional recognition method and the deep learning recognition method. The traditional recognition methods mainly include logistic regression (LR) (Bootkrajang and Kabán, 2012), K-nearest neighbors (KNN) (Mucherino et al., 2009), support vector machine (SVM) (Hearst et al., 1998), and decision tree (DT) (Fürnkranz, 2010) algorithm. The deep learning recognition methods are represented by the second-generation ANNs, mainly including the AlexNet (Krizhevsky et al., 2012), GoogleNet (Szegedy et al., 2015), MobileNet (Howard et al., 2017), DenseNet (Huang et al., 2017), and ResNet (He et al., 2016). Among them, the ResNet adopts the ResNet-18, ResNet-34, and ResNet-50 models with the different layers.

To better evaluate the model effect in different methods, this study uses the precision, recall, and F1-score as the evaluation metrics, where F1-score can be defined by precision and recall. The recognition result for the target category *i* can be expressed by:


(19) $$\begin{array}{l} \text{Precisio}{\text{n}}_{i}=\frac{TP}{TP+FP} \end{array}$$

(20) $$\begin{array}{l} \text{Recal}{\text{l}}_{i}=\frac{TP}{TP+FN} \end{array}$$

(21) F1i=2×Precisioni×RecalliPrecisioni+Recalli

where, *TP* represents the number of positive samples that are correctly recognized, *FP* represents the number of negative sample that is misrecognized as the positive sample, and *FN* represents the number of positive sample that is misrecognized as the negative sample. Considering the imbalance of the various targets in the SAR-Dataset, to measure the performance of the model more accurately, this study uses the weighted average of the ship recognition results of each category as the final performance of the model. Therefore, the weighted result of each category can be expressed by:


(22) $$\begin{array}{l} \text{Precision}=\underset{i}{\sum }\frac{N_{i}}{N}\text{Precisio}{\text{n}}_{i} \end{array}$$

(23) $$\begin{array}{l} \text{Recall}=\underset{i}{\sum }\frac{N_{i}}{N}\text{Recal}{\text{l}}_{i} \end{array}$$

(24) $$\begin{array}{l} \text{F1}=\underset{i}{\sum }\frac{N_{i}}{N}\text{F}{\text{1}}_{i} \end{array}$$

where, *N*_i_ is the number of target samples with category *i* and *N* is the sample size of the entire dataset.

This SAR-Dataset dataset has been divided as follows: 75% is the training set, 25% is the test set, and 20% is divided from the training set as the validation set. The training set is used to train the model, the validation set is used to adjust the model parameters and select the optimal weight of the model, and the test set is used to test the performance of the model. The validation and test sets are not visible during the model training. For the traditional recognition method, the principal component analysis (PCA) method is used to reduce the dimension of the input SAR image, and the image features are extracted as the input to predict the category, where the feature dimension is set to 80. For the neural network model, Adam is used as an optimizer for the training, where the training batch size is 32, the learning rate is 0.001, the weight decay is set to 0.0001, and the loss function is the cross-entropy loss. A total of 100 rounds of training are performed, and the optimal weight of the model is saved according to the results on the validation set. For the proposed method in this study, the membrane time constant τ is set to 2, the simulation duration *T* is set to 12, the loss function is the mean square error function, and the rest of the settings are consistent with the neural network model in the comparison method. The experimental hardware adopts a computer with the CPU as the Intel i9-9900X, GPU as the NVIDIA RTX 2080 Ti, and the operating system is Ubuntu18.04. For the traditional method, it is implemented using the Scikit-learn (Swami and Jain, 2013) framework. For the neural network model, it is implemented using the PyTorch (Paszke et al., 2019) deep learning framework, and CUDA10.1 is used for the acceleration during the training and testing.

### Experimental results and analysis

#### Ship recognition results

Table 1 shows the ship recognition result of the different models on the SAR-Dataset. The results are presented in the form of percentages (%), and the recognition results with the best performance in each indicator are shown in bold. It can be observed the accuracy, recall, and F1 scores of each model are relatively similar; thus the three indicators can well-measure the ship recognition performance of the models. From the perspective of three indicators, the traditional recognition methods are not as good as the neural network method based on deep learning, and the F1 score of the best performing KNN algorithm is only 92.5%. This is because the traditional target recognition method has the problem of the weak adaptive ability. If the SAR image to be recognized has large defects or other external noise interference, the model cannot obtain the ideal recognition result. However, the SAR images usually have a lot of noise, and only using the surface features of the SAR images (such as the PCA features) cannot fully extract the information contained in the SAR images, which leads to being easily affected by the background clutter or coherent speckle noise. On the contrary, the deep learning-based neural network methods extract the SAR image features through the convolution layer and uses the gradient descent algorithm for the iterative training optimization, so it can more accurately recognize the ships in the SAR image, and the noise in the SAR image can be avoided to a certain extent through the adaptive learning of the convolution kernel weight. According to the experimental result based on the SAR-Dataset, the effectiveness of the CNN for the SAR image recognition has been fully demonstrated.

**Table 1:** Ship recognition results of different models.

**Methods**	**Models**	**Precision (%)**	**Recall (%)**	**F1 (%)**
Traditional recognition method	LR (Bootkrajang and Kabán, 2012)	92.67	92.65	92.58
	KNN (Mucherino et al., 2009)	92.50	92.49	92.50
	SVM (Hearst et al., 1998)	88.13	87.37	87.75
	DT (Fürnkranz, 2010)	89.34	89.39	89.36
Deep learning recognition method	AlexNet (Krizhevsky et al., 2012)	92.67	92.73	92.68
	GoogleNet (Szegedy et al., 2015)	94.44	94.49	94.46
	MobileNet (Howard et al., 2017)	94.81	94.82	94.80
	DenseNet (Huang et al., 2017)	95.14	95.15	95.15
	ResNet-18 (He et al., 2016)	95.05	95.07	95.06
	ResNet-34 (He et al., 2016)	94.92	94.90	94.96
	ResNet-50 (He et al., 2016)	94.10	94.06	94.05
	**Proposed SpikingSAR model**	**95.57**	**95.59**	**95.58**

The experimental results with the best performance are shown in bold.

For the different CNN models, there are also great differences in their performance. The early CNN like the AlexNet has performed poorly, with the F1 scores of only 92.68%. This is due to the shallow number of the model layers, which cannot extract well the high-level features in the images; thus, it is easily affected by the SAR image noise. Compared with AlexNet, the GoogleNet network deepens the network depth and improves the recognition performance. However, the CNN is prone to the problem of gradient disappearance when the number of the model layers is deepened, which makes the model difficult to train. Thus, the DenseNet, ResNet, and MobileNet alleviate the problem of gradient disappearance by introducing the residual structure, which can achieve better recognition performance. In addition, it can also be found that with the deepening of the number of the layers of the ResNet, the recognition effect of the model has declined to a certain extent. The above experiment results show that a certain depth of the neural network can better extract the SAR image features and reduce the noise interference. However, when the number of the model layers is greatly deepened, since the SAR images do not have the rich features compared to the optical images, the deeper network structures may lead to poor model performance.

The proposed SpikingSAR model adopts a combination architecture of the CNN and SNN networks. To reduce the complexity of the model and avoid the problems caused by the deep network, it adopts a shallow structure design, which can achieve the best ship recognition performance based on the SAR-Dataset with an F1 score of 95.58%. The results in Table 1 fully demonstrate the effectiveness of the proposed SpikingSAR method. All evaluation indicators outperform the traditional ship recognition methods, and in most cases achieve the comparable or even better results than the CNN methods.

Table 2 shows the confusion matrix of the ship recognition using the proposed SpikingSAR method. The confusion matrix is often used for the recognition and classification tasks in the supervised learning, which can visually display the prediction results of the model for the different categories. From the results in Table 2, it is seen that the proposed SpikingSAR method can identify the ship samples relatively accurately, and only a few other samples have false alarms. At the same time, due to the high similarity between the ship-like samples and background samples, the proposed SpikingSAR method may misidentify the two in a few cases.

**Table 2:** Confusion matrix of the ship recognition results using the proposed SpikingSAR.

	**Ship sample**	**Ship-like sample**	**Background sample**	**F1 (%)**
Ship sample	**396**	2	1	98.55
Ship-like sample	2	**377**	20	93.31
Background sample	1	24	**374**	94.21
Weighted F1 (%)				**95.58**

The experimental results with the best performance are shown in bold.

**Figure 8:**
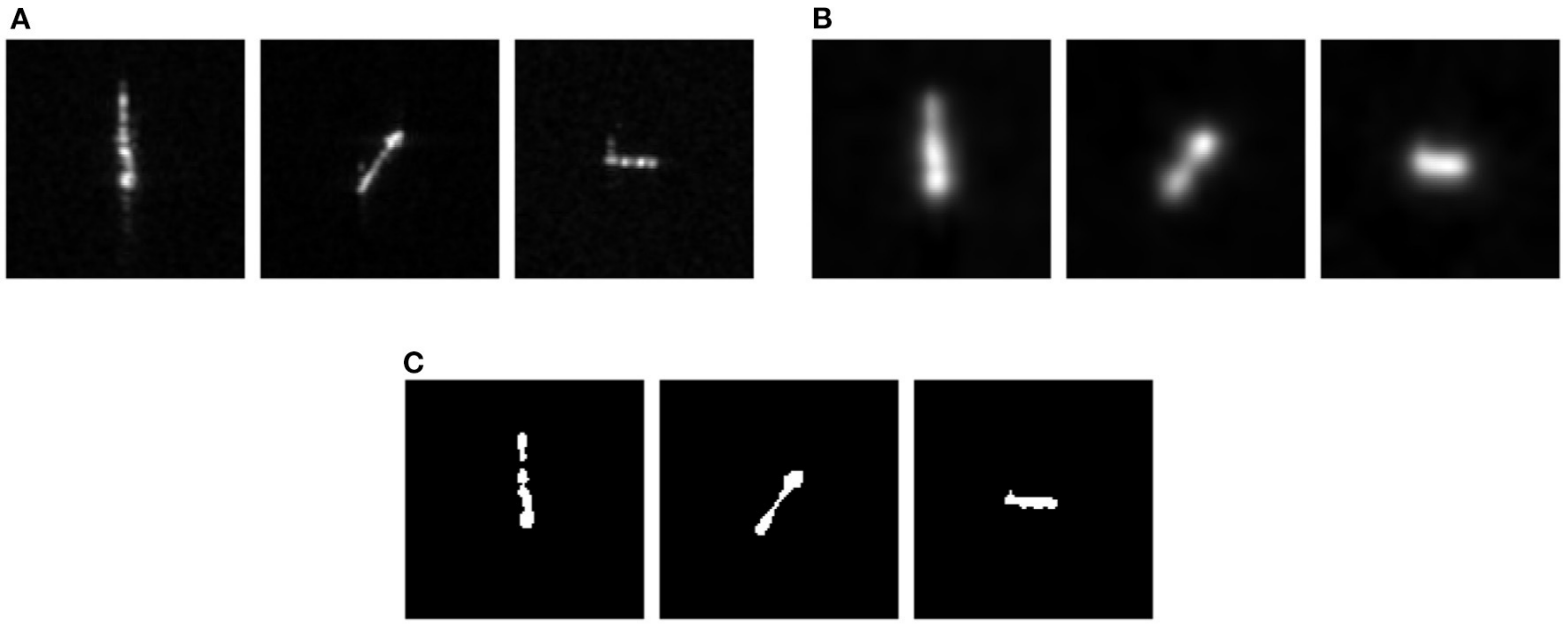
**(A)** SAR ship image samples; **(B)** Feature saliency map; **(C)** Spike coding map.

Figure 8 shows the SAR ship image samples, the corresponding feature saliency map, and spike coding map. According to Figure 8B, it can be observed that the feature saliency map based on the visual attention mechanism can better describe the ship outline in the input SAR images, and then eliminate the influence of the ocean background clutter, so that the target area is in the visually more obvious. Figure 8C is the spike encoding image obtained by using the feature saliency map through the Poisson encoder. Since the spike is a discrete sequence of 0 and 1, the encoded visual saliency map is a binary image. The encoded binary image can well-extract the ship in the SAR images, so it can improve the learning of the salient features of the SAR image for the proposed SpikingSAR model and improve the robustness and anti-interference of the model to the noise. Thus, it is very necessary to use the attention mechanism in the proposed SpikingSAR method, which can further improve the accuracy of the ship recognition the SAR image.

#### Model parameter comparison

**Figure 9:**
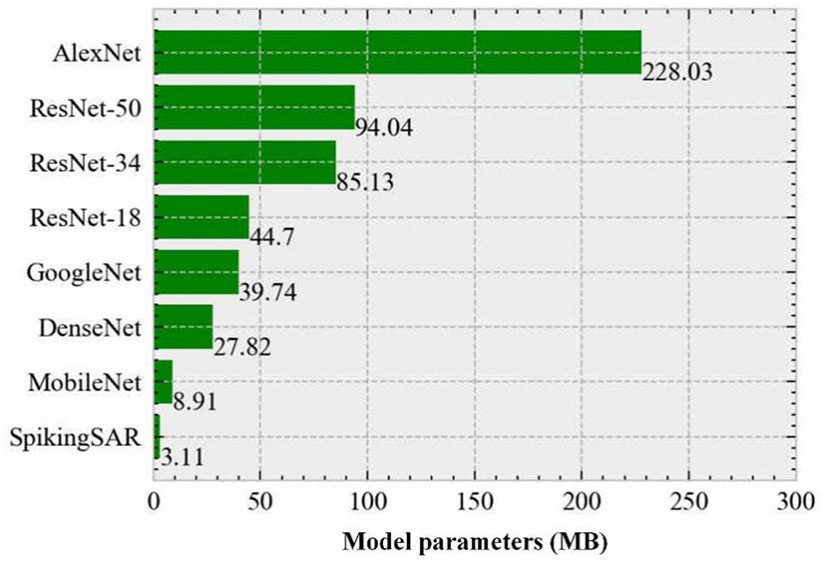
Parameter comparison of the different neural network models.

Although with the development of the deep learning theory and the continuous breakthrough in the computing speed of the hardware devices, the CNN has higher and higher accuracy in various image processing tasks, the model and network structure are becoming more and more complex, which inevitably brings the problem of a huge amount of the model parameters. Figure 9 gives the parameter comparison of the different neural network models. As shown in Figure 9, the number of parameters of the AlexNet model reaches 228.03 MB. This is because the design of the shallow structure of the AlexNet relies on the extraction and recognition of the image features by subsequent fully connected layers; thus, too many fully connected layers lead to large model parameters. GoogleNet and ResNet adopt the design of the stacked convolutional layers in the model structure, and only use the fully connected layer in the last layer of the model, thus greatly reducing the amount of the model parameters. To reduce the model size while maintaining the model performance, MobileNet introduces a depth separable convolution operation, which decomposes one layer of the convolution into two layers of the computation and obtains the same output with fewer parameters and computation, further reducing the model parameters. Compared with the above-mentioned CNN, DenseNet is a more lightweight model, which can improve the information and gradient flow of the entire network by connecting all layers in the network to each other, and greatly reduces the number of the channel of the convolutional layer. Therefore, DenseNet can achieve better performance with fewer parameters.

From Figure 9, it can be found that the parameter amount of the proposed SpikingSAR model is the smallest among all models (only 3.11 MB), which is far less than the model parameters of the AlexNet and is also less than that of the optimized MobileNet and DenseNet. The small number of parameters of the SpikingSAR model comes from the following two aspects. First, there is an LIF layer without the parameters. SpikingSAR uses a combination of the convolution layer and LIF spiking neuron layer. The main function of the LIF layer is to simulate the charging and discharging process of the spiking neurons, which does not contain trainable parameters. Second, there is a shallow network structure design. Because the spike coding based on the visual attention mechanism is performed on the SAR image at the input layer, the image feature information is enhanced; thus, the high-order information of the image can be effectively extracted without a deep network structure. In summary, the SpikingSAR has the less model parameters, which reduces the memory resources required in the operation process and is helpful for porting and deploying the terminal devices in actual scenarios.

#### Model energy consumption

To further explore the energy efficiency of the proposed method, this study uses the appropriate indicators to measure the number of the operations of the SpikingSAR and contrasting neural networks and then calculates their energy consumption. The calculation of energy consumption is closely related to the number of operands in the hardware, and the calculation methods of the operands are different for the different neural networks. For the traditional ANNs running on the modern GPUs, the indicator to measure the number of the operations is mainly the floating-point operations (FLOPs), which can be considered as an indicator to measure the computational performance of the model. For the SNN based on brain-like intelligence, since it uses the spiking neurons to transmit the signals between synapses, the indicator to measure its operation number is mainly the number of synaptic operations (SOPs), which is mainly the voltage changes of the neuron membrane, specifically the number of t voltage changes during the neuron charging and discharging.

Considering the possible deviation of the energy-consumption calculation caused by different hardware characteristics, this study follows the quantitative method (Wu et al., 2019) to calculate the energy consumption of each model and assumes that all models are running on appropriate equipment. Specifically, the traditional ANNs run on the Intel Stratix 10 TX FPGA, which is one of the most energy-efficient computing platforms with an energy-consumption of 12.5pJ/FLOP. The SNNs run on the neuromorphic hardware ROLLS (Indiveri et al., 2015), which can provide the efficient calculation of trigger events by transmitting the spike signals between neurons, and only consumes the computing resources during the process of neurons firing spikes, and the energy consumption is 77fJ/SOP.

Table 3 shows the comparison of operations and energy consumption of different neural network models. From Table 3, it is found that the SpikingSAR based on the spike signal transmission has fewer operands with the SOPs 17.97M, which is only 1/30–1/2 times of the operands of the other ANNs, illustrating the efficiency of the process of the transmitting signals using the spikes. Furthermore, using the SpikingSAR on the neuromorphic hardware ROLLS consumes much less energy than traditional ANNs according to the energy-consumption values of the individual methods. Finally, the SpikingSAR achieves nearly three orders of the magnitude energy efficiency advantage, which fully demonstrates the significant energy efficiency of the proposed SpikingSAR method.

**Table 3:** Comparison of the operations (FLOPs/SOPs) and energy consumption for different models.

**Models**	**Operations (FLOPs/SOPs)**	**Energy-consumption**
AlexNet (Krizhevsky et al., 2012)	98.59M	1.23 × 10^−3^J
GoogleNet (Szegedy et al., 2015)	143.07M	1.79 × 10^−3^J
MobileNet (Howard et al., 2017)	43.54M	0.54 × 10^−3^J
DenseNet (Huang et al., 2017)	291.63M	3.65 × 10^−3^J
ResNet-18 (He et al., 2016)	243.53M	3.04 × 10^−3^J
ResNet-34 (He et al., 2016)	489.96M	6.12 × 10^−3^J
ResNet-50 (He et al., 2016)	549.02M	6.86 × 10^−3^J
**Proposed SpikingSAR model**	**17.97M**	**1.38** **× 10**^**−6**^**J**

The experimental results with the best performance are shown in bold.

#### Applicability analysis

The calculation of the SpikingSAR method in each forward propagation includes the propagation of the spike train in the network and the update of the neuron state variables. The calculation time of the spike propagation is mainly determined by the length and quantity of the transmitted spike train, while the calculation time of the neuron state variable update is mainly determined by the spike neuron model. Since the length of the spike train in this experiment is fixed as the number of the pixel values encoded in the input image, the calculation of the spike train propagation in the network is mainly affected by the number of spikes, which is determined by the selected simulation duration.

The simulation duration *T* is one of the most important parameters for the time-driven SNN models, but its value is often difficult to determine. When the value of *T* is too small, the computational complexity and energy consumption can be reduced, but it cannot accurately describe the changing process of the neuron states. When the value of *T* is too large, it will increase the amount of the network computation and energy-consumption, and it may also destroy the network simulation accuracy of the spike signals. The selection of the spiking neuron model is crucial to the performance of the SNN. A suitable neuron model can use a reasonable mathematical model to the intelligent computing process of the biological systems, and integrate biologically inspired, efficient, and accurate neural information processing mechanisms to the SNN. The SNN is given better biological credibility and strong fitting ability.

**Figure 10:**
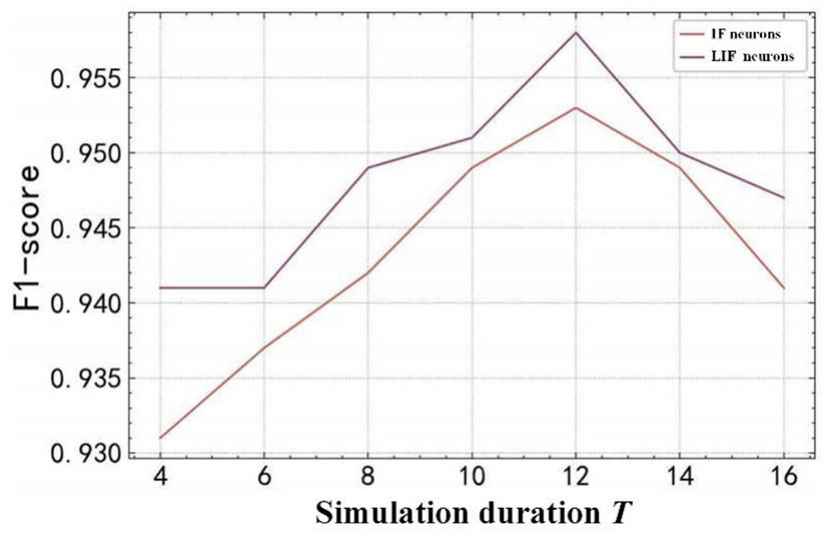
Comparison of different neuron models and simulation duration.

The experimental analysis is conducted for different simulation durations *T* and spiking neuron models, which is shown in Figure 10. In Figure 10, it can be observed that: (1) The different spiking neuron models have different performances. The LIF neuron model has outperformed the IF neuron model in terms of F1 score, because the LIF model additionally considered the key features of leakage in the process of biological neuron membrane potential changes in the process of simplifying the neuron state changes, which can describe the details of the neuron activity more accurately, thereby enhancing the biological accuracy and performance of the SNN. (2) SpikingSAR model is sensitive to different values of *T*. When *T* is small, the SpikingSAR using the IF neuron and LIF neuron models performs poorly. When *T* increases, the simulation time step becomes finer, and then the recognition accuracy of the SpikingSAR increases gradually. The peak value is reached when *T*=12, indicating that *T*=12 is a more reasonable choice of the simulation duration. When *T* is larger than 12, the recognition accuracy of the SpikingSAR begins to decline, which indicates that a large *T* will affect the recognition performance of the model. The possible reason for this phenomenon is that the large *T* leads to too many input spike trains, and the error accumulation caused by the training method using the surrogate gradient gradually increases, which finally affects the learning ability of the model.

In summary, for the proposed SpikingSAR method, using the LIF neuron model is a more reasonable choice. In addition, considering that the model is greatly affected by the value of *T*, it is necessary to select an appropriate value of *T* according to the actual situation during the experiment, so that the model can not only accurately simulate the voltage changes of the neurons, but also minimize the amount of computation and energy consumption while maintaining the optimal model performance.

## Conclusion

This study presents a high-efficiency and low-energy ship recognition strategy based on the SNN in the SAR images. First, a Poisson encoder based on the visual attention mechanism is used to encode the input SAR image with a spike train, which can remove the background noise during the encoding process and preserve the visual saliency of the image as much as possible. Then, based on the LIF neuron model and combined with the CNN, an end-to-end SNN model has been constructed, which uses the LIF neuron spike firing the frequency to perform the ship recognition in the SAR images. Finally, to solve the problem that the SNN model is difficult to train, the Arctan function is used as a surrogate function for the spike emission function during the backpropagation for the gradient calculation, and the backpropagation algorithm is applied to the training process of the SpikingSAR. The experiments have been conducted based on the SAR-Dataset in real scenarios, which are used to compare and analyze the traditional methods and mainstream deep learning methods. The experimental results show that the proposed SpikingSAR can accurately recognize the ships, and then has achieved a good performance in various evaluation indicators. Compared with the comparison methods, it has the advantages of fewer parameters, high efficiency, and low energy consumption, which is very conducive to the deployment of the terminal equipment with the high energy-efficiency. In the future works, we would like to use some newly released ship target recognition datasets for the ship recognition in the SAR images. In addition, some recent ship recognition methods (He et al., 2021; Lang et al., 2022) will be used to compare the approaches.

## Data availability statement

The original contributions presented in the study are included in the article/supplementary material, further inquiries can be directed to the corresponding author/s.

## Author contributions

HX: experiments, research methods, data processing, writing the original draft, and funding acquisition. GW: writing the original draft, resources, supervision, project administration, and funding acquisition. KX and XJ: guided experiments. XH and ZW: performed the writing—review. All authors have contributed to the paper and approved the submitted version.

## Funding

This work was co-supported by the Guangdong Basic and Applied Basic Research Foundation (No. 2021A1515010768), the University Stability Support Program Project of Shenzhen (No. 2022502), the National Natural Science Foundation of China (No. 62201566), the Natural Science Foundation of Guangdong (No. 202214050002344), and the Fundamental Research Funds for the Central Universities, Sun Yat-sen University (No. 2022ZZ028).

## Conflict of interest

The authors declare that the research was conducted in the absence of any commercial or financial relationships that could be construed as a potential conflict of interest.

## Publisher's note

All claims expressed in this article are solely those of the authors and do not necessarily represent those of their affiliated organizations, or those of the publisher, the editors and the reviewers. Any product that may be evaluated in this article, or claim that may be made by its manufacturer, is not guaranteed or endorsed by the publisher.
